# Positive and negative affects in university students at the beginning of the COVID-19 pandemic and a year after it was announced

**DOI:** 10.1192/j.eurpsy.2022.1293

**Published:** 2022-09-01

**Authors:** L. Shaigerova, O. Almazova, O. Vakhantseva, R. Shilko

**Affiliations:** Lomonosov Moscow State University, Psychology, Moscow, Russian Federation

**Keywords:** university students, Covid-19, mental health, positive and negative affects

## Abstract

**Introduction:**

The emotional state of university students is critical for their successful learning, efficient interacting with people around, and increasing the quality of life in general. The COVID-19 pandemic widely affected the mood and emotional state of student youth.

**Objectives:**

The study focuses on tracing the dynamics of positive and negative affects among students in the first few weeks after the announcement of the COVID-19 pandemic and one year later.

**Methods:**

Positive and Negative Affect Schedule (PANAS, Watson, Clark, Tellegen, 1988) was applied in the research. The study involved 210 university students aged 18 to 23. The study was conducted online shortly after the COVID-19 pandemic was declared in spring 2020 (N = 105) and a year later (winter-spring 2021) (N=105). Given that the sizes of subsamples are comparable ANOVA was used for the periods under consideration (Levene Statistic > 0.05).

**Results:**

One-way analysis of variance ANOVA showed that evaluations of positive affect differ significantly (p <0.05) while estimations of negative affect do not differ (p> 0.05) during the periods of the COVID-19 pandemic (spring 2020 and winter-spring 2021 ). With Post Hoc Scheffe, it was also shown that the positive affect scores in spring 2020 were significantly higher than in winter-spring 2021 (p <0.05).

**Conclusions:**

Thus, it has been shown that although the level of negative affect in students did not increase a year after the beginning of the COVID-19 pandemic, the level of positive emotions significantly decreased during this period. The reported study was funded by RFBR, project number 20-04-60174.

**Disclosure:**

No significant relationships.

